# Procedure Proposal for Minimising the Dynamic Error of Second-Order Sensors

**DOI:** 10.3390/s22051901

**Published:** 2022-02-28

**Authors:** Krzysztof Tomczyk, Małgorzata Kowalczyk, Ksenia Ostrowska

**Affiliations:** 1 Faculty of Electrical and Computer Engineering, Cracow University of Technology, Warszawska 24, 31-155 Krakow, Poland; 2 Faculty of Mechanical Engineering, Cracow University of Technology, Jana Pawła II 37 Avenue, 31-864 Krakow, Poland; kowalczyk@mech.pk.edu.pl (M.K.); ksenia.ostrowska@pk.edu.pl (K.O.)

**Keywords:** dynamic error, measurement accuracy, second-order sensors

## Abstract

This paper proposes the procedure for minimising the dynamic error in the time and frequency domains, based on the example of a second-order sensor. Our procedure includes three main steps: modelling of the sensors using the Monte Carlo (MC) method; determination of the maximum value of the dynamic error using the integral-square criterion (ISC); and optimisation of the parameters of the sensor model by minimising the ISC. The uncertainties associated with the modelling procedure and the MC method are also considered. The mathematical formulae necessary for implementation in a given programming language (MathCad, MATLAB, C, etc.) are presented in detail. The proposed procedure was implemented in the frequency domain, using MathCad 15, and applied to the example of the Althen 731-207 accelerometer. Validation of the proposed procedure was carried out using a digital signal processor of type TMS320C6713. The proposed procedure can increase the accuracy of the signal processing obtained at the output of sensors applied to a wide range of measurements.

## 1. Introduction

The min–max method has been applied to minimise the dynamic error of a wide class of measurement systems [1,2] and can be used for the optimisation of both single- and multi-criteria systems [3]. Application of the min–max procedure yields satisfactory results by first considering the upper bound on the dynamic error (UBDE) (the max stage of the min–max algorithm) [4,5,6], and then by applying a selected procedure for minimising this criterion (the min stage) [7,8]. Two basic quality criteria are typically used for the dynamic measurement: i.e., the integral-square criterion (ISC) [7,9] and the absolute error criterion [5].

The UBDE is the maximum value of the dynamic error and depends on both the dynamic properties of the sensor and the shape of the input signal [7]. It is determined by computer simulation based on the mathematical model of the sensor [10,11,12] and the test signal used at the input. The shapes most often considered are linearly and parabolically increasing signals [13], a random Gaussian process [14], or a specially defined function of time [15]. However, such signals do not reflect the real inputs to the sensors.

A mapping of the realistic shapes of the input signals can be obtained when test signals with constraints are used [7,16]; however, this approach requires the use of special computational procedures, including tools such as algorithms to solve the system of convolutional equations (after their optimal number has been determined) [17], procedures based on the genetic method [17,18], and procedures using the fixed-point algorithm (FPA) [9,19]. However, the influence of the uncertainty [20,21] associated with the sensor modelling on the value of the dynamic error is not considered in these procedures.

At the min stage, there are many possibilities for the selection of the dedicated algorithm. Procedures based on the genetic method and the Levenberg-Marquardt algorithm [17,18] have been developed for the UBDE step. However, the influence of the modelling uncertainty on the obtained result, and the propagation of uncertainties by the corresponding computational procedure, have not been considered [21,22].

In view of the limitations of the min–max procedure, we propose the procedure based on the FPA and the Monte Carlo (MC) method [23,24,25,26,27]. Although our procedure involves minimising the dynamic error for second-order sensors [28], it can easily be extended to the sensors with dynamics of different orders. We chose to focus on second-order sensors in this paper because a significant proportion of real sensors are defined by this dynamic order, such as vibration and pressure sensors [28,29,30,31,32], and the mechanical construction of a wide variety of other measurement instruments [33]. The proposed procedure can be applied in both the time and frequency domains [34,35,36], which are used in practical implementations of procedures intended for sensor modelling. The solutions proposed in this study consists of three main steps:

1. Modelling of the sensors using the MC method. In addition to the parameters of the sensor mathematical model under consideration, the uncertainties associated with these parameters are also considered [37,38,39]. Pseudo-random number generators with normal and uniform distributions are used to implement the MC method [40,41,42], which has an advantage over other alternative procedures as it can be implemented in both the time and frequency domains.
2. Determination of the UBDE for the ISC by imposing magnitude and time constraints on the input test signal [7,17]. FPA, which is highly computationally effective, is used here to determine the UBDE [9,19].
3. Optimisation of the sensor parameters using the MC method. The min–max procedure proposed in this paper allows us to select the parameters of the second-order sensors in such a way as to obtain the minimum value of the UBDE. For obvious reasons, the corresponding parameters are optimised while maintaining the bandwidth of the sensor.

This new approach to sensors modelling can significantly increase the accuracy of processing dynamic signals (by several percent) [43]. In addition, the reliability of the obtained measurement results increases when the uncertainties associated with the sensor parameters are taken into consideration.

**Figure 1:**
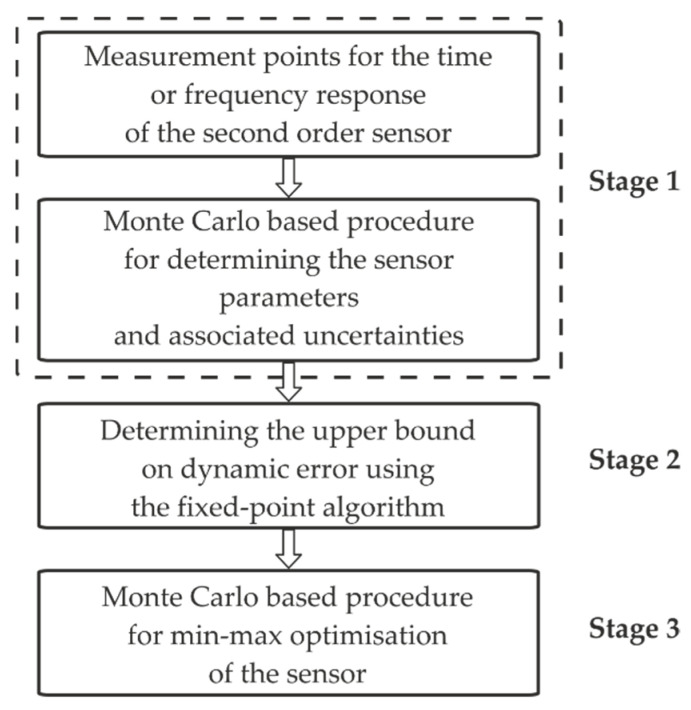
Block diagram of the procedure for minimising the dynamic error.

Figure 1 shows the block diagram of the procedure for minimising the dynamic error introduced by the sensors. The proposed procedure consists of three main stages. The first stage involves the synthesis of the time or frequency response for the second-order sensor and the MC-based procedure for determining the corresponding parameters and associated uncertainties. The methods for synthesising the measurement points for the corresponding responses are not considered here. Computer-aided methods using a measurement data acquisition card and the control and measurement software, such as LabVIEW [44,45], are generally used for this purpose.

Section 3 is devoted to the synthesis of mathematical models of the second-order sensors. Our MC-based procedure for determining the parameters and associated uncertainties forms the original contribution of this study.

The second stage involves the determination of the UBDE using the FPA. This algorithm is based on the step responses of the considered sensor and corresponding reference, which forms a comparison model for the determination of the UBDE. The procedure used to calculate the UBDE with the FPA is discussed in Section 4.

The third stage is our MC-based procedure for min–max optimisation of the sensor. Predetermined ranges are considered for each parameter of the mathematical model of the sensor. The parameter values for the sensor model are determined based on these ranges, finally resulting in the minimal value of the UBDE. The proposed MC-based procedure for minimising the dynamic error is discussed in detail in Section 5.

The transfer function for second-order sensors is represented by the following formula:

(1) $$K\left(s\right)=\frac{a\omega_{0}{}^{2}}{s^{2}+2\beta \omega_{0}s+\omega_{0}{}^{2}}$$

where f is the frequency [Hz], a is the static sensitivity coefficient [V], β is the damping factor (dimensionless), and $\omega_{0}=2\pi f_{0}$, where $f_{0}$ is the natural undamped frequency [Hz].

The inverse Laplace transformation, after multiplying by 1/s, gives the following step response:

(2) $$h\left(t\right)=a\{1-\exp\left(-\omega_{0}\beta t\right)[\cos\left(\omega_{0}\sqrt{1-\beta^{2}}t\right)+\frac{\beta }{\sqrt{1-\beta^{2}}}\sin\left(\omega_{0}\sqrt{1-\beta^{2}}t\right)]\}.$$

In the time domain, the parameters β and $\omega_{0}$ are represented by the following formulas [28]:

(3) $$\beta =\frac{\ln\left(\frac{\Delta y}{a}\right)}{\sqrt{\ln^{2}\left(\frac{\Delta y}{a}\right)+\pi^{2}}}$$

and

(4) $$\omega_{0}=2\pi f_{0}=\frac{2\pi }{T_{n}\sqrt{1-\frac{\ln^{2}\left(\frac{\Delta y}{a}\right)}{\ln^{2}\left(\frac{\Delta y}{a}\right)+\pi^{2}}}},$$

where Δy and $T_{n}$ denote the overshoot [V] and period of the damped vibrations [s], respectively [34]. Equations (3) and (4) are used to apply the MC method in the time domain.

The amplitude is determined as the modulus based on the complex frequency response K(jω), which corresponds to the transfer function represented by Equation (1). The amplitude response is expressed as follows:

(5) $$\left|K\left(j\omega \right)\right|=A\left(\omega \right)=\frac{a}{\sqrt{{\left(1-\frac{\omega^{2}}{\omega_{0}{}^{2}}\right)}^{2}+{\left(\frac{2\beta \omega }{\omega_{0}}\right)}^{2}}}.$$

In the frequency domain, the parameters β and $\omega_{0}$ are defined as follows:

(6) $$\beta =\frac{\;\sqrt{M_{r}-\sqrt{M_{r}{}^{2}-a^{2}}}}{\sqrt{2\cdot M_{r}}}$$

and

(7) $$\omega_{0}=\frac{\omega_{r}}{\sqrt{1-\frac{M_{r}-\sqrt{M_{r}{}^{2}-a^{2}}}{M_{r}}}},$$

where $M_{r}$ denotes the resonant peak [V], and

(8) $$\omega_{r}=2\pi f_{r}=\omega_{0}\;\sqrt{1-2\beta^{2}},\;0<\beta <1/\sqrt{2},$$

where $f_{r}$ denotes the resonant frequency [Hz] [35,36].

Equations (7) and (8) form the basis for applying the MC method in the frequency domain.

## 2. Procedure for Modelling the Second Order Sensors Using the Monte Carlo Method

The modelling procedure for the second-order sensors in the time domain can be divided into several stages, as described below.

**Stage 1:** The vector of parameters

(9) $$W=\left[a^{\mathrm{int}},\;\Delta y^{\mathrm{int}},T_{n}{}^{\mathrm{int}}\right]$$

is intuitively determined for the time vector

(10) $$t=\left[t_{0},t_{1},\ldots ,\;t_{N-1}\right]$$

based on the measurement points of the step response

(11) $$h=\left[h_{0},h_{1},\ldots ,\;h_{N-1}\right],\;n=0,1,\ldots ,N,$$

where N denotes the number of measurement points [23].

The vector in Equation (9) is determined for the assumed parametric model

(12) $$h\left(t\right)=f\left(t,W\right)=\;=a^{\mathrm{int}}\{1-\exp\left[\frac{-2\pi }{T_{n}{}^{\mathrm{int}}\sqrt{1-\frac{\ln^{2}\left(\frac{\Delta y^{\mathrm{int}}}{a^{\mathrm{int}}}\right)}{\ln^{2}\left(\frac{\Delta y^{\mathrm{int}}}{a^{\mathrm{int}}}\right)+\pi^{2}}}}\frac{-\ln\left(\frac{\Delta y^{\mathrm{int}}}{a^{\mathrm{int}}}\right)}{\sqrt{\ln^{2}\left(\frac{\Delta y^{\mathrm{int}}}{a^{\mathrm{int}}}\right)+\pi^{2}}}t\right][\cos\left(\frac{2\pi }{T_{n}{}^{\mathrm{int}}}t\right)+\;+\frac{T_{n}{}^{\mathrm{int}}}{T_{n}{}^{\mathrm{int}}\sqrt{1-\frac{\ln^{2}\left(\frac{\Delta y^{\mathrm{int}}}{a^{\mathrm{int}}}\right)}{\ln^{2}\left(\frac{\Delta y^{\mathrm{int}}}{a^{\mathrm{int}}}\right)+\pi^{2}}}}\frac{-ln\left(\frac{\Delta y^{\mathrm{int}}}{a^{\mathrm{int}}}\right)}{\sqrt{\ln^{2}\left(\frac{\Delta y^{\mathrm{int}}}{a^{\mathrm{int}}}\right)+\pi^{2}}}\sin\left(\frac{2\pi }{T_{n}{}^{\mathrm{int}}}t\right)]\}.$$

**Stage 2:** Determination of the number of MC draws M. The lowest number of draws is determined using the following formula:

(13) $$M>10^{4}/\left(1-p\right),$$

based on the guidelines included in [37,38], where p is the assumed confidence level and is generally set to 0.95. The successive draws are denoted by m=0,1,…,M−1.

**Figure 2:**
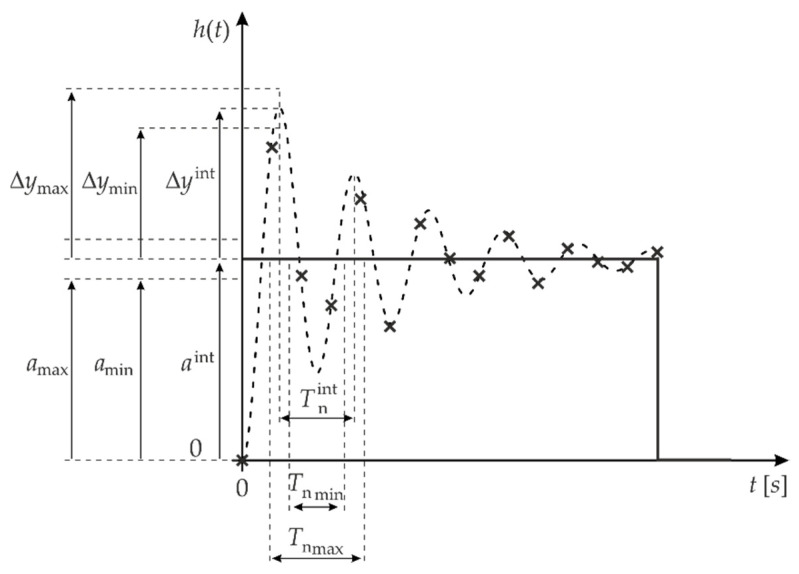
Example of determining the draw ranges for parameters a, Δy, and $T_{n}$.

**Stage 3:** Determination of the draw ranges for the parameters a, Δy, and $T_{n}$, which should be symmetric around the values of the following parameters: $a^{\mathrm{int}},\;\Delta y^{\mathrm{int}}$, and $T_{n}{}^{\mathrm{int}}$ (Figure 2). For the determined draw ranges, we expect that there will be parameters of the model given by Equation (1) for which the minimum value of the regression error can be obtained. The values of the parameters that minimise the modelling error are denoted as $\tilde{a},$ $\Delta \tilde{y}$ and ${\tilde{T}}_{n}.$

**Stage 4:** Selection of the type of pseudo-random number generator.

**Figure 3:**
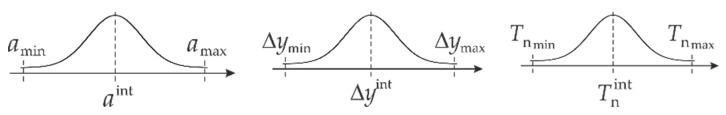
Application of the generator with a normal distribution for the execution of MC draws.

Based on Figure 2, it can be assumed that for the model in Equation (1), with parameter values close to $a^{\mathrm{int}},\;\Delta y^{\mathrm{int}}$, and $T_{n}{}^{\mathrm{int}}$, there is the greatest probability of obtaining the parameters:$\;\tilde{a},$ $\Delta \tilde{y}$, and ${\tilde{T}}_{n}.$ It, therefore, seems reasonable to choose a generator with a normal distribution [41], as shown in Figure 3.

We denote the function executing the pseudo-random number generator by prng, and estimates of the parameters: a, Δy and $T_{n}$ by the symbols: $\hat{a},$ $\Delta \hat{y}$ and ${\hat{T}}_{n}.$ The following equations show how the function prng is used:

(14) $$\hat{a}=\;\mathrm{prng}\left(M,a_{\min},a_{\max}\right),$$

(15) $$\Delta \hat{y}=\;\mathrm{prng}\left(M,\Delta y_{\min},\Delta y_{\max}\right)$$

and

(16) $${\hat{T}}_{n}=\;\mathrm{prng}\left(M,\;T_{n}{}_{\min},T_{n}{}_{\max}\right).$$

**Stage 5:** Determination of the matrix **Φ** based on the randomly selected values M for the parameters: $\hat{a},$ $\Delta \hat{y}$, and ${\hat{T}}_{n}.$ The matrix **Φ** is obtained by substituting the above parameters into the model of the step response h(t) in Equation (12) for the particular values of the vector **t**. The matrix **Φ** is given by the formula:

(17) $$\Phi =\left[\begin{array}{ccc} {\tilde{h}}_{0,0} & \ldots & {\tilde{h}}_{0,M-1}\\ \vdots & \ddots & \vdots \\ {\tilde{h}}_{N-1,0} & \ldots & {\tilde{h}}_{N-1,M-1} \end{array}\right].$$

**Stage 6:** Determination of the matrix **Δ** associated with the model error. This matrix is determined by subtracting the transposed vector in Equation (11) from the particular columns in the matrix given by Equation (17). We then have:

(18) $$\Delta =\left[\begin{array}{ccc} {\tilde{h}}_{0,0}-h_{0} & \ldots & {\tilde{h}}_{0,M-1}-h_{0}\\ \vdots & \ddots & \vdots \\ {\tilde{h}}_{N-1,0}-h_{N-1} & \ldots & {\tilde{h}}_{N-1,M-1}-h_{N-1} \end{array}\right].$$

**Stage 7:** Determination of the vector representing the sum of the squared errors for each column of the matrix Δ. This vector has the following form:

(19) $$S=\sum_{m}\Delta^{2}.\;$$

**Stage 8:** Determination of the minimum value $S_{\min}$ of the vector S and the corresponding drawing number $m_{\min}$. The parameters: $\tilde{a},$ $\Delta \tilde{y}$, and ${\tilde{T}}_{n}\;$ resulting from the value $S_{\min}$ are assumed to represent the optimal solution to the regression task executed based on the corresponding measurement points.

**Stage 9:** The modelling error is calculated for the particular measurement points

(20) $$\Delta {\mathrm{apr}}_{n}=\Delta_{n,m_{\min}}.$$

**Stage 10:** Determination of the uncertainty of regression using the MC method:

(21) $$u\left(\mathrm{MC}\right)=\sqrt{\frac{1}{M\left(M-1\right)}\sum_{m=0}^{M-1}{\left(S_{m}-\bar{S}\right)}^{2}},$$

where

(22) $$\bar{S}=\frac{1}{M}\sum_{m=0}^{M-1}S_{m}.$$

**Stage 11:** Determination of the uncertainties associated with the parameters: $\tilde{a},$ $\Delta \tilde{y}$, and ${\tilde{T}}_{n}$ using the following formula:

(23) $$u\left({\tilde{x}}_{1}\right)=\sqrt{\frac{1}{M\left(M-1\right)}\sum_{m=0}^{M-1}{\left({\hat{x}}_{1m}-{\bar{x}}_{1}\right)}^{2},}$$

where $x_{1\mathrm{opt}}$ denotes the corresponding optimal parameters, and the mean [38] is given by the simple formula:

(24) $${\bar{x}}_{1}=\frac{1}{M}\sum_{m=0}^{M-1}{\hat{x}}_{1m}.$$

The modelling procedure for second-order sensors in the frequency domain can be divided into the following stages [27]:

**Stage 1:** Substitution of Equations (6) and (7) into Equation (5), to give the function $A\left(\omega ,a,\;M_{r},\omega_{r}\right).$

**Figure 4:**
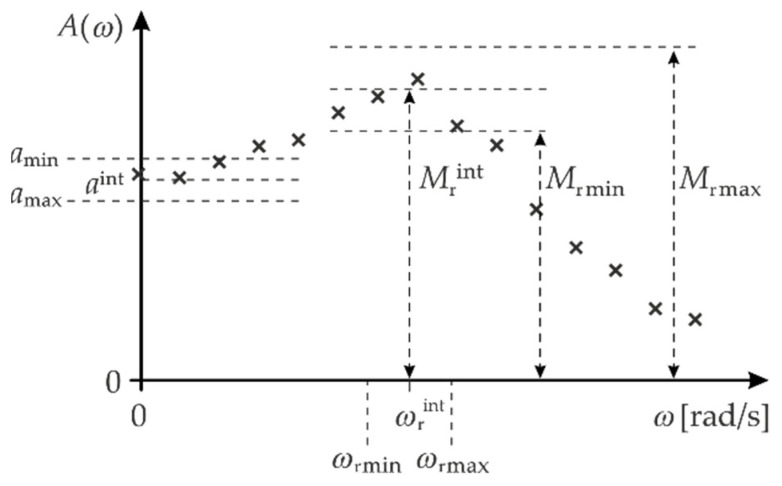
Example of determining of the draw ranges for parameters a, $M_{r}$ and $\omega_{r}$.

**Stage 2:** Intuitive estimation of the approximate values of the parameters: $a^{\mathrm{int}},$ $M_{r}^{\mathrm{int}}$, and $\omega_{r}^{\mathrm{int}}$ based on the measurement points for the amplitude response (Figure 4).

**Stage 3:** Determination of the number of MC draws M. The minimum value of M is calculated using Equation (11).

**Stage 4:** Determination of the draw ranges for the parameters: a, $\;M_{r}$, and $\omega_{r}$, which should be symmetric around the parameters: $a^{\mathrm{int}},$ $M_{r}^{\mathrm{int}}$, and $\omega_{r}^{\mathrm{int}}$ (Figure 4). The parameter values that minimise the error are denoted below by the symbols: $\tilde{a},$ ${\tilde{M}}_{r}$, and ${\tilde{\omega }}_{r}.$

**Stage 5:** Selection of a pseudo-random number generator.

The ranges of the MC draws for the parameters: $a^{\mathrm{int}},$ $M_{r}^{\mathrm{int}}$, and $\omega_{r}^{\mathrm{int}}$ are determined in an analogous way, as shown in Figure 3.

**Stage 6:** Determination of the matrix:

(25) $$\Psi =\left[\begin{array}{ccc} A{\left(\omega_{0}\right)}_{0} & \ldots & A{\left(\omega_{0}\right)}_{M-1}\\ \vdots & \ddots & \vdots \\ A{\left(\omega_{M-1}\right)}_{0} & \ldots & A{\left(\omega_{N-1}\right)}_{M-1} \end{array}\right],$$

where N is the number of measurement points of the amplitude response A(ω). The matrix Ψ is determined by substituting the parameters: $a^{m},$ $M_{r}{}^{m}$ and $\omega_{r}{}^{m}$ into Equation (5) for the particular items $\omega_{n},$ where n=0, 1,…, N−1. The parameters: $a^{m},$ $M_{r}{}^{m}$, and $\omega_{r}{}^{m}$ are obtained during the successive MC draws: m=0, 1, …, M−1.

**Stage 7:** Determination of the matrix corresponding to the modelling error, using the formula:

(26) $$\Delta =\left[\begin{array}{ccc} A{\left(\omega_{0}\right)}_{0} & \ldots & A{\left(\omega_{0}\right)}_{M-1}\\ \vdots & \ddots & \vdots \\ A{\left(\omega_{N-1}\right)}_{0} & \ldots & A{\left(\omega_{N-1}\right)}_{M-1} \end{array}\right],$$

where $\tilde{A}{\left(\omega_{n}\right)}_{m}=A{\left(\omega_{n}\right)}_{m}-A\left(\omega_{n}\right)$. The subsequent rows of the matrix **Δ** reflect the modelling error for the particular pulsations $\omega_{n}$.

**Stage 8:** Determination of the following vector

(27) $${\mathrm{Sk}}^{\Delta }=\sum_{m}\Delta^{2},\;$$

where the elements are the sum of the squared errors for each column of the matrix Δ.

**Stage 9:** Determination of the minimum value of the parameter $Sk_{\min}^{\Delta },$ based on the vector $Sk^{\Delta }$ and the corresponding draw number $m_{\min}$. The parameters: $\tilde{a},$ ${\tilde{M}}_{r}$, and ${\tilde{\omega }}_{r},$ which correspond to the value of the parameter $Sk_{\min}^{\Delta }$, are assumed to be the optimal solutions for the regression of the sensor amplitude response.

**Stage 10:** Determination of the parameters: $\tilde{\beta }$ and ${\tilde{\omega }}_{0},$ using Equations (7) and (8), respectively.

**Stage 11:** Determination of the regression uncertainty using the MC method, according to the following formula:

(28) $$u\left(\mathrm{MC}\right)=\sqrt{\frac{1}{M\left(M-1\right)}\sum_{m=0}^{M-1}{\left({\mathrm{Sk}}_{m}^{\Delta }-{\bar{\mathrm{Sk}}}^{\Delta }\right)}^{2}},\;$$

where the mean is

(29) $${\bar{\mathrm{Sk}}}^{\Delta }=\frac{1}{M}\sum_{m=0}^{M-1}{\mathrm{Sk}}_{m}^{\Delta }$$

**Stage 12:** Determination of the uncertainties: $u\left(\tilde{a}\right),$ $u\left({\tilde{M}}_{r}\right)$, and $u\left({\tilde{\omega }}_{r}\right)$ for the particular parameters: $\tilde{a},$ ${\tilde{M}}_{r}$, and ${\tilde{\omega }}_{r},$ using the formula:

(30) $$u\left({\tilde{x}}_{2}\right)=\sqrt{\frac{1}{M\left(M-1\right)}\sum_{m=0}^{M-1}{\left({\hat{x}}_{2m}-{\bar{x}}_{2}\right)}^{2},}$$

where

(31) $${\bar{x}}_{2}=\frac{1}{M}\sum_{m=0}^{M-1}{\hat{x}}_{2m},$$

and ${\tilde{x}}_{2}$ corresponds to the particular sensor parameters.

**Stage 13:** Determination of the complex uncertainties: $u\left(\tilde{\beta }\right)$ and $u\left({\tilde{\omega }}_{0}\right),$ associated with the parameters: $\tilde{\beta }$ and ${\tilde{\omega }}_{0},$ based on the following formulae:

(32) $$u\left(\tilde{\beta }\right)=\sqrt{{\left[\frac{\partial \beta }{\partial a}u\left(\tilde{a}\right)\right]}^{2}+{\left[\frac{\partial \beta }{\partial M_{r}}u\left({\tilde{M}}_{r}\right)\right]}^{2}}$$

and

(33) $$u\left({\tilde{\omega }}_{0}\right)=\sqrt{{\left[\frac{\partial \omega_{0}}{\partial a}u\left(\tilde{a}\right)\right]}^{2}+{\left[\frac{\partial \omega_{0}}{\partial M_{r}}u\left({\tilde{M}}_{r}\right)\right]}^{2}+{\left[\frac{\partial \omega_{0}}{\partial \omega_{r}}u\left({\tilde{\omega }}_{0}\right)\right]}^{2}},$$

where the partial derivatives: $\frac{\partial \beta }{\partial a}$ and $\frac{\partial \beta }{\partial M_{r}}$ are determined using Equation (6), and the derivatives: $\frac{\partial \omega_{0}}{\partial a},$ $\frac{\partial \omega_{0}}{\partial M_{r}}$, and $\frac{\partial \omega_{0}}{\partial \omega_{r}}$ are calculated using Equation (7) [38].

## 3. Determining the UBDE Using the FPA

Taking into account the test signal constrained in the magnitude and time [5,9,16], and the integral-square criterion (ISC), the UBDE is defined as follows:

(34) $$\mathrm{UBDE}=\int_{0}^{T}\left[\int_{0}^{T}H_{t}\left(t-\tau \right)x_{0}\left(\tau \right)d\tau \right]x_{0}\left(t\right)dt,$$

where t∈(0,T), while $x_{0}\left(t\right)$ and T denote the constrained test signal. The function $H_{t}\left(t\right)$ is defined, as follows:

(35) $$H_{t}\left(t\right)=\int_{0}^{T}\frac{d}{dt}h_{t}\left(t-v\right)\frac{d}{dt}h_{t}\left(v-\tau \right)dv,$$

where $h_{t}\left(t\right)$ and T denote the total step response and time for the steady state of the sensor [9]. The above test signal produces the UBDE which is defined by Equation (34). The total step response is given by the formula:

(36) $$h_{t}\left(t\right)=h\left(t\right)-h_{r}\left(t\right),$$

where h(t) is defined in Equation (2), while $h_{r}\left(t\right)$ denotes the step response of the reference used to determine the UBDE. A simple approach is to use a low-pass filter with the bandwidth corresponding to the operating range of the considered sensor [7,18]. The order of the filter should be as high as possible. However, it is limited by the computational possibilities of the mathematical software used for the corresponding calculations.

The value of time T is selected to be equal to the steady state of the total step response.

In order to calculate the UBDE according to Equation (34), it is necessary to determine the signal $x_{0}\left(t\right)$. This signal is obtained using the FPA, as follows:

1. Calculate the function $H_{t}\left(t\right)$ according to Equation (35).
2. Determine the initial signal
  (37) $$x^{0}\left(t\right)=M\cdot \mathrm{sgn}\left[H_{t}\left(t\right)\right],$$
  where M denotes the magnitude constraint, and the symbol sgn corresponds to the signum function.
3. Determine the i+1  input signal
  (38) $$x^{i+1}\left(t\right)=M\cdot \mathrm{sgn}\left[x^{i}\left(\tau \right)H_{t}\left(\tau -t\right)d\tau \right]$$
  for i=0, 1,…, I, where I denotes the number of iterations selected in advance [9,19].

The final solution, denoted by the symbol: $x^{I}\left(t\right)$ corresponds to the signal $x_{0}\left(t\right)$, which is included in Equation (34). The signal $x_{0}\left(t\right)$ is the basis for determination of the UBDE. To correct the UBDE by the value resulting from the uncertainties: $u\left(\tilde{a}\right),$ $u\left(\tilde{\beta }\right)$, and $u\left({\tilde{\omega }}_{0}\right),$ it is necessary to consider the following ranges defined by a uniform distribution: $\tilde{a}\pm u\left(\tilde{a}\right),$ $\tilde{\beta }\pm u\left(\tilde{\beta }\right)$ and ${\tilde{\omega }}_{0}\pm u\left({\tilde{\omega }}_{0}\right)$. For this purpose, the MC method is applied as described in Section 3, except that a pseudo-random number generator with a uniform distribution is used. The corrected UBDE value is denoted below in the following as ${\mathrm{UBDE}}^{c}$. The parameters corresponding to this error are denoted by the symbols: $a^{c},$ $\beta^{c}$, and $\omega_{0}{}^{c}$, while the associated uncertainties are denoted as $u(a^{c}),$ $u(\beta^{c})$, and $u(\omega_{0}{}^{c})$.

## 4. Monte Carlo Based Procedure for Minimising the Error

When we have obtained the parameters: $a^{c},$ $\beta^{c}$, $\omega_{0}{}^{c}$ and the associated uncertainties: $u(a^{c}),$ $u(\beta^{c})$, and $u(\omega_{0}{}^{c})$ for the corresponding ${\mathrm{UBDE}}^{c},$ it is then possible to implement the min–max optimisation procedure. This procedure is carried out to determine these parameters for the sensor model for their predetermined ranges of variability. The minimum value of the UBDE, defined by Equation (34), can be obtained for these ranges. This value is denoted below in the following as UBDE^min^. We assume a uniform distribution for the particular ranges of parameters involved in the optimisation procedure. For these defined ranges, we use the MC method to determine the value of the parameters, which minimise the UBDE according to the ISC.

**Figure 5:**
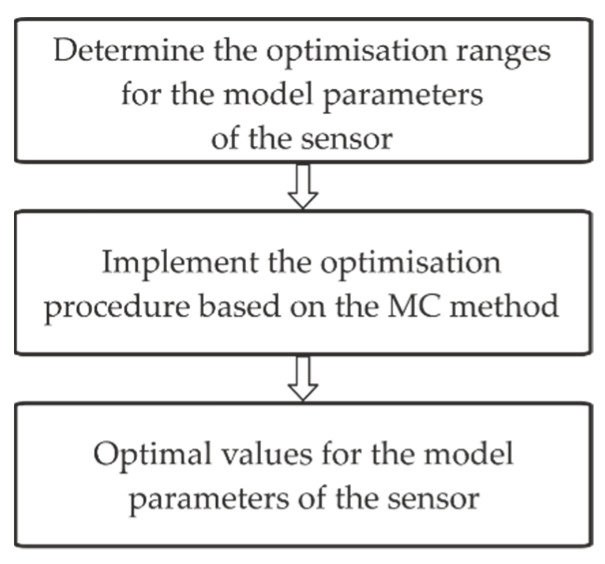
Block diagram for the optimisation procedure.

Figure 5 shows the block diagram for the proposed optimisation procedure.

The parameter ranges are assumed in advance in the first stage of the proposed procedure. An optimisation is performed in the second stage by applying the MC method, based on a pseudo-random number generator with a uniform distribution. We use the notations $a^{\mathrm{opt}},$ $\beta^{\mathrm{opt}}$, and $\omega_{0}{}^{\mathrm{opt}}$ for the parameters of the second-order sensor that are obtained as a result of applying the optimisation procedure. The uncertainties associated with these parameters are denoted as follows: $u(a^{\mathrm{opt}}),$ $u(\beta^{\mathrm{opt}})$, and $u\left(\omega_{0}{}^{\mathrm{opt}}\right)$. The lowest possible value of the error, defined by Equation (34), can be obtained using the optimal values of the sensor parameters. The highest value of the accuracy for the considered sensor corresponds to the lowest error determined in this way.

## 5. Materials and Methods

In this section, we describe the application of the proposed procedure in the frequency domain to the example of the Althen 731-207 accelerometer. Intuitive values for the MC method are set to $a^{\mathrm{int}}=1\;V/{\mathrm{ms}}^{-2},$ $M_{r}^{\mathrm{int}}=2\;V/{\mathrm{ms}}^{-2}$, and $f_{r}^{\mathrm{int}}=2.5\;\mathrm{kHz}$ (Figure 4). These values were obtained based on the measurement points of the amplitude response. Then, the minimum and maximum range for the deviation of the above parameters are established as follows: $a_{\min}=0.9$ $V/{\mathrm{ms}}^{-2},$ $a_{\max}=1.1$ $V/{\mathrm{ms}}^{-2},$ $M_{r}{}_{\min}=1.7$ $V/{\mathrm{ms}}^{-2},$ $M_{r}{}_{\max}=2.3$ $V/{\mathrm{ms}}^{-2},$ $f_{r}{}_{\min}=2.0$ kHz, and $f_{r}{}_{\max}=3.0$ kHz. 

To determine the parameters: $\tilde{a},$ ${\tilde{M}}_{r}$ and ${\tilde{f}}_{r}$, a normally distributed pseudo-random number generator (a Box-Muller generator) was used. A number of MC trials equal to $2\times 10^{5}$ was carried out, and the MC and the following results were obtained from the simulation: ${\mathrm{Sk}}_{\min}^{\Delta }=0.08581\;V/{\mathrm{ms}}^{-2},$ $m_{\min}=138134,$ $\tilde{a}=0.9814$ $V/{\mathrm{ms}}^{-2},\;\;\;{\tilde{M}}_{r}=1.972\;V/{\mathrm{ms}}^{-2}$, and ${\tilde{f}}_{r}=2292\;\mathrm{Hz}.$ Then, using Equations (6) and (7), the following values were calculated: $\tilde{\beta }=0.2512$ and ${\tilde{f}}_{0}=2469$ Hz. The uncertainty u(MC), determined using Equation (27), was equal to 0.02318 $V/{\mathrm{ms}}^{-2}$, while the uncertainties were obtained using Equation (31) as $u\left(\tilde{a}\right)=11\times 10^{-4}$ $V/{\mathrm{ms}}^{-2},$ $u\left({\tilde{M}}_{r}\right)=$ $15\times 10^{-3}$ $V/{\mathrm{ms}}^{-2}$ and $u\left({\tilde{f}}_{r}\right)=14\;\mathrm{Hz}.$ The complex uncertainties $u\left(\tilde{\beta }\right)$ and $u\left({\tilde{f}}_{0}\right)$ were obtained using Equations (32) and (33) as $14\times 10^{-4}$ and 11 Hz, respectively.

The parameters: $a^{c},$ $\beta^{c}$, $f_{0}{}^{c}$ and the associated uncertainties: $u(a^{c}),$ $u(\beta^{c})$, $u(f_{0}{}^{c})$ were determined for the ranges: $\tilde{a}\pm u\left(\tilde{a}\right),$ $\tilde{\beta }\pm u\left(\tilde{\beta }\;\right)$ and ${\tilde{f}}_{0}\pm u\left({\tilde{f}}_{0}\right),$ corresponding to a uniform distribution. A tenth-order Butterworth filter was applied as a reference to determine the ${\mathrm{UBDE}}^{c}$ [19,27]. The time T corresponding to the steady state of the total step response $h_{t}\left(t\right)$ was equal to 5 ms. Based on the results of applying our MC method and the FPA, the ${\mathrm{UBDE}}^{c}$ was obtained as equal to $18.3\;{\mathrm{mV}}^{2}s$. The corresponding accelerometer parameters and associated uncertainties were as follows: $a^{c}=0.9816\;V/{\mathrm{ms}}^{-2},$ $\beta^{c}=0.2509,$ $f_{0}{}^{c}=2462\;\mathrm{Hz}$,$\;u(a^{c})=18\times 10^{-4}$ $V/{\mathrm{ms}}^{-2},$ $u(\beta^{c})=20\times 10^{-4}$ and $u(f_{0}{}^{c})=13\;\mathrm{Hz}$.

The last step of the proposed procedure involves the optimisation process described in Section 5. The optimisation ranges for the parameters:$\;\beta^{c}$ and $f_{0}{}^{c}$ were determined in relation to the maximum tolerance of the parameter $a^{c}$, which was set to the range ±10%. Based on the amplitude response, given by Equation (6), the following ranges: (0.230–0.270) and (2468–2696) were obtained for the parameters: $\beta^{c}$ and $f_{0}{}^{c}$, respectively. The value of the parameter a was assumed to be equal to the value of parameter $a^{c},$ since it is evident that as this parameter increases, the UBDE also increases. The results of the min–max optimisation process were obtained as follows: $\beta^{\mathrm{opt}}=0.270$ and $f_{0}{}^{\mathrm{opt}}=2544\;\mathrm{Hz}.$ Finally, the UBDE^min^ was found to be equal to $17.8\;{\mathrm{mV}}^{2}s$, while the associated uncertainties were as follows: $u(\beta^{\mathrm{opt}})=17\times 10^{-4}$ and $u\left(f_{0}{}^{\mathrm{opt}}\right)=9\;\mathrm{Hz}$.

Our results indicate that the value of UBDE increases as the parameter β is increased. A comparison of the UBDE^min^ ($17.8\;{\mathrm{mV}}^{2}s$) and ${\mathrm{UBDE}}^{c}$ ($18.3\;{\mathrm{mV}}^{2}s$) demonstrates the influence of the min–max optimisation on the error UBDE. This influence is seen in a reduction in the UBDE of 2.73% as a result of applying the optimisation procedure to the Althen 731-207 accelerometer. The reduction in UBDE corresponds to the increase in the dynamic accuracy of the accelerometer.

## 6. Validation of the Proposed Procedure

The proposed procedure and the results presented in Section 5 were validated using a digital signal processor (DSP) of type TMS320C6713 [46]. This processor was programmed in the C language to follow the numerical procedures presented in Section 2, Section 3 and Section 4. The use of DSP made it possible to process the measurement points in real time, and both the time and frequency characteristics were obtained.

**Figure 6:**
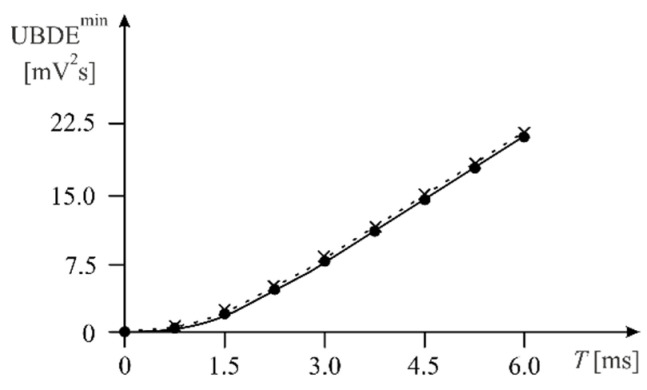
Relationship between the UBDE^min^ and the time *T* for the Althen 731-207 accelerometer testing; MathCad (solid line), DSP (dotted line).

Figure 6 shows the relationship between the UBDE^min^ and the testing time T∈(0, 6.0 ms) for the Althen 731-207 accelerometer, obtained using MathCad 15 (solid line) and DSP (dotted line).

Figure 6 shows that when DSP was used, slightly higher (approx. 2%) UBDE^min^ values were obtained. The tests were carried out for varying values of the time T, which was increased in steps of 0.75 s.

This difference is due to the numerical calculation methods used in MathCad and in DSP (implemented in the C programming language). However, it should be stated that the results obtained with DSP are very approximated with the results obtained with the computer simulation. It should also be noted that the use of DSP allows the proposed min–max optimisation to be implemented in real time, i.e., by combining a practical experiment that allows us to determine time or frequency characteristics with the numerical calculations.

## 7. Conclusions

The results presented in Section 6 from modelling the Althen 731-207 accelerometer confirm that the proposed procedure can be effectively applied to both scientific and engineering applications. Our procedure involves the use of two numerical methods: FPA and MC. The first is an iterative algorithm with a high degree of convergence and computational efficiency, while the second one is recommended by JCGM guides and can be straightforwardly implemented using mathematical and computational programs (MathCad, MATLAB, C, etc.). The most important finding is that the proposed procedure can be used for modelling in both the time and frequency domains.

The solutions presented in this paper for second-order sensors can be extended to sensors with other dynamic orders; however, this requires the derivation of the relevant mathematical formulae which describe the time and frequency responses of the sensor. In addition to the transfer function used in this study as the basis for deriving the mathematical formulae in the time and frequency domains, it is also possible to use a description of the sensor using a state observer.

The procedure presented in this paper can be implemented using a digital signal processor, although this would require the integral equations presented in Section 3 to be converted to their digital counterparts. In addition, it would be necessary to convert the source code of the algorithm described in Section 2 to codes appropriate to the digital signal processor.

## Data Availability

Not applicable.
